# DRD4 allele frequencies in greylag geese vary between urban and rural sites

**DOI:** 10.1002/ece3.9811

**Published:** 2023-02-08

**Authors:** Sabrina Mai, Caroline Wittor, Stefan Merker, Friederike Woog

**Affiliations:** ^1^ Department of Zoology State Museum of Natural History Stuttgart Stuttgart Germany; ^2^ Center of Excellence for Biodiversity and integrative Taxonomy University of Hohenheim Stuttgart Germany

**Keywords:** *Anser anser*, behavior/social evolution, candidate gene, flight initiation distance, greylag geese, urbanization

## Abstract

With the increasing urbanization of the last decades, more and more bird species occur in urban habitats. Birds which thrive in urban habitats often have a higher tolerance toward human disturbance and show behaviors which differ from their rural counterparts. There is increasing evidence that many behaviors have a genetic basis. One candidate gene is the dopamine receptor D4 (DRD4), which has been associated with fear and thus, flight initiation distance (FID). In this study, we analyzed a segment of DRD4 in greylag geese *Anser anser*, describing the variability of this gene across several geographically distant populations, and comparing its variability between an urban and a rural site in south–west Germany. We additionally measured FIDs of urban and rural geese to test for a possible correlation with DRD4 genotypes. We found a high variation within DRD4, with 10 variable sites leading to 11 alleles and 35 genotypes. Two genotypes occurred in 60% of all geese and were thus defined as common genotypes versus 33 rare genotypes. Population differentiation was very low between the urban and rural sites in Germany but common genotypes occurred more often in the urban area and rare genotypes more often in the rural area. FID was significantly higher at the rural site, but no significant correlation between FID and DRD4 genotypes could be detected. Nevertheless, our results suggest that local site selection may be related to DRD4 genotypes.

## Graphical abstract

There is increasing evidence that the dopamine receptor D4 (DRD4) influences fear and thus, flight initiation distance (FID). We compared greylag geese living in an urban and a rural site and found that common DRD4 genotypes occurred more often in urban sites while rare genotypes occurred more often in the rural site. FID was significantly higher at the rural site, but no significant correlation between FID and DRD4 genotypes could be detected.

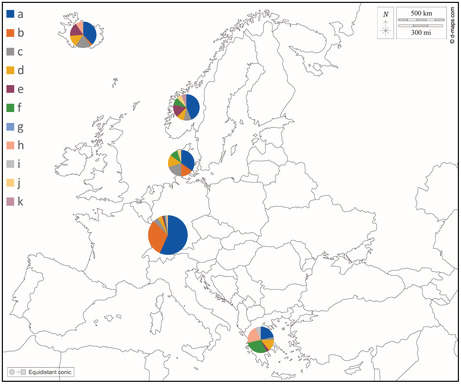

## 1 INTRODUCTION

Over the last decades, urbanization has been increasing globally, with the majority of humans (55%) now living in urban areas (United Nations, 2019). This leads to large‐scale modifications of natural habitats (Batáry et al., 2018), with negative impacts on some wildlife and native biodiversity (McKinney, 2008; Peterson et al., 2007). However, urban habitats also provide new opportunities for many species, for example due to the heterogeneity of the habitat and the increased availability of food, such as via bird feeders (McKinney, 2008). Birds living in urban habitats are faced with near‐continuous anthropogenic disturbance, through pedestrian or vehicular traffic, artificial lights, and the increased noise level (Lowry et al., 2013). However, urban environments can also have positive effects on avian wildlife, in particular due to supplementary feeding, the generally higher abundance of food sources and higher temperatures in winter (Amrhein, 2013; Murray Mitchell, 1961; Tratalos et al., 2007). There are many species which seem to thrive well in urban environments, often occurring in large numbers (Lowry et al., 2013; Sol et al., 2013). For example, house sparrows (*Passer domesticus*) mainly occur in urban areas (Carrete & Tella, 2011; De Laet & Summers‐Smith, 2007) and some species that used to live exclusively in natural habitats such as European blackbirds (*Turdus merula*; Luniak et al., 1990) or American crows (*Corvus brachyrhynchos*; Withey & Marzluff, 2005) have since colonized cities.

A higher tolerance toward anthropogenic disturbance is necessary for colonizing and living in urban habitats (Møller, 2009). Different environmental conditions may foster different phenotypes based on the same genotype, which is called phenotypic plasticity (Kelly et al., 2012). This is often reflected through behavioral flexibility (i.e., the ability to adapt a behavior to the environment), such as a bird's reaction to fear and thus its tolerance toward disturbance (Sol et al., 2002, 2013; Sol & Lefebvre, 2000; Thibert‐Plante & Hendry, 2011).

However, there is a tendency that the behavioral variation of a single individual can be lower than the variation among the individuals in the population (Carrete & Tella, 2017). This consistent behavior of individuals, such as bold versus shy individuals, is often called animal personality (Carrete & Tella, 2017; Dall et al., 2004). High fear thresholds may indicate boldness (i.e., risk‐prone behavior; Blumstein, 2006; Wilson et al., 1994) and birds with higher fear thresholds are likely to be more successful in urban areas (Carrete & Tella, 2011; Møller, 2009). These fear thresholds are not necessarily related to stress hormone levels (as recently shown for reptiles and birds; Injaian et al., 2020) but may depend on the animal's personality (Carrete & Tella, 2017; Dall et al., 2004). If birds with high fear thresholds are more successful, a selection on boldness in urban areas may occur (Møller, 2009). Alternatively, there might be a pre‐establishment selection, where only bold animals disperse into urban areas (Carrete et al., 2012; Chapple et al., 2012).

The phenotypic variation of behaviors can also be based on genotypic differences (van Oers & Mueller, 2010): In a meta‐analysis, van Oers and Sinn (2013) extracted data from 75 studies and found that there is sufficient evidence for a genetic inheritance of personality traits. One candidate gene for personality variation is the dopamine receptor D4 (DRD4; Savitz & Ramesar, 2004). The neurotransmitter dopamine regulates many functions in the vertebrate central nervous system (Callier et al., 2003) and its receptor D4 is an important component of the dopaminergic system (Savitz & Ramesar, 2004). Dopamine in general and DRD4 specifically have been associated with temperament and behaviors such as novelty seeking in a variety of organisms (e.g., horses *Equus caballus* in Momozawa et al., 2005, dogs *Canis familiaris* in Hejjas et al., 2007; for an overview see Inoue‐Murayama, 2009; Savitz & Ramesar, 2004). In mice (*Mus musculus*), the knock‐out of DRD4 led to individuals with a lower behavioral response to novelty (Dulawa et al., 1999; Falzone et al., 2002) and in vervet monkeys (*Cercopithecus aethiops*) one variant of DRD4 was associated with novelty seeking (Bailey et al., 2007). The most frequently studied species is the great tit (*Parus major*), where a single SNP (single nucleotide polymorphism) in the DRD4 gene has been associated with exploratory behavior and novelty seeking (Fidler et al., 2007; Riyahi et al., 2017; Timm et al., 2015, 2019 but see also Korsten et al., 2010). A significant association between the DRD4 genotype and neophobia/neophilia (novelty avoidance/seeking) was also found in yellow‐crowned bishops (*Euplectes afer*; Mueller et al., 2014) and collared flycatchers (*Ficedula albicollis*; Garamszegi et al., 2014). Collared flycatchers with specific DRD4 genotypes also showed lower risk‐taking behavior (Garamszegi et al., 2014). In blue tits (*Cyanistes caeruleus*), a different single SNP was associated with escape behavior (Kluen et al., 2012). However, studies in common starlings (*Sturnus vulgaris*) and Seychelles warbler (*Acrocephalus sechellensis*) found no correlation between DRD4 and behavior (Edwards et al., 2015; Rollins et al., 2015).

Another personality trait that has been associated with DRD4 is shyness or boldness. To measure this trait, an individual's reaction toward an approaching threat can be used. Using flight initiation distance (FID) to detect wariness is a well‐established method (Blumstein, 2006; Carrete & Tella, 2010; Holtmann et al., 2016). FID is the distance between an animal and a potential threat at which the animal begins to flee. In field studies, approaching humans can act as the potential threat in a standardized manner (Blumstein, 2006). When the FID of an animal is low, its low wariness indicates a bolder personality (Scales et al., 2011). FID has been correlated to DRD4 genotypes in dunnocks (*Prunella modularis*; Holtmann et al., 2016) and black swans (*Cygnus atratus*; van Dongen et al., 2015). In dunnocks, there are significant associations between DRD4 polymorphisms and FID (Holtmann et al., 2016), while in black swans, wary individuals were associated with rare genotypes and the rural location (van Dongen et al., 2015). van Dongen et al. (2015) compared FIDs and genotypes of an urban and a rural population, as urban animals often show behaviors which differ from their rural counterparts (Møller, 2009). They used extensive FID data, but only a small number of genotyped animals (*n* = 80). They acknowledged a lack in statistical power to test for differences between different genotypes and thus grouped genotypes depending on their frequency into rare or common (van Dongen et al., 2015).

In our study, we aimed to comprehensively analyze a large dataset for genotypic data to test whether DRD4 can be linked to wariness in a new species. If so, we add to the growing body of evidence for a genetic basis for behavior. Additionally, by comparing urban and rural locations, we aim to gain insight on the influence of human activities regarding the behavior and possibly the genetic makeup of our study species. Our study species is the greylag goose (*Anser anser*), which occurs in autochthonous populations across Europe (Fox & Leafloor, 2018). In south‐west Germany, greylag geese live in the city of Stuttgart and average between 200 and 300 individuals since 2010 (Mai et al., 2022). Many studies have looked at social behavior in greylag geese, making them an excellent model organism for behavioral analyses (Scheiber et al., 2019).

The aims of this study are (1) to describe the variability of the DRD4 gene across autochthonous populations of greylag geese in Iceland, Norway, Denmark, and Greece and (2) to test the correlation of this variability with an ecological factor (urbanization) in a local population in Germany. We hypothesize that DRD4 genotype frequencies differ between rural and urban areas and predict that genotypes carried by bolder animals predominate at the urban sites. To test for variation in boldness, (3) we compared FID data between the urban and the rural site and predict that the genotype frequencies will correlate with FIDs.

## 2 MATERIALS AND METHODS

### 2.1 Study species and study area

Greylag geese (*Anser anser*; *Anatidae, Anseriformes*) are herbivorous birds with two subspecies: the nominate western greylag goose (*A. a. anser*) and the eastern greylag goose (*A. a. rubirostris*; del Hoyo et al., 1992). Greylag geese can be found in Europe and Asia, with increasing numbers (Fox & Leafloor, 2018). In Germany, both subspecies as well as hybrids can be found. Greylag geese are classified as regionally established and occur both in autochthonous and introduced populations (Bauer et al., 2016).

To get an initial idea of the variation of the DRD4 gene in greylag geese and to avoid sampling bias (Morin et al., 2004) we analyzed samples from autochthonous breeding populations of *A. a. anser* in Iceland (Icelandic flyway; Powolny et al., 2018), Norway and Denmark (Atlantic flyway; Powolny et al., 2018) and a sedentary population of *A. a. rubrirostris* in Greece, Prespa (Bounas et al., 2018). Greylag geese from Iceland winter in the United Kingdom, while Norwegian and Danish greylag geese winter in the Netherlands and Spain, respectively. After the breeding season, they are widely hunted (Madsen et al., 1999).

All samples from Iceland and Norway were collected from hunted geese following national hunting regulations. Danish samples were provided by the Copenhagen Museum (see accession numbers in Sample Information file uploaded to DataDryad) and Grecian samples were provided by the Society for the Preservation of Prespa.

In our study area in Stuttgart, south‐west Germany, greylag geese use public parks for feeding and roosting. Greylag geese were first seen at the Max‐Eyth‐Lake in the early 1980s, with a wild‐type breeding pair which likely escaped from captivity (Hölzinger et al., 2004). They first bred successfully in 1995 and the population has since expanded to 200–300 individuals (Mai et al., 2022). These geese are non‐migratory and remain in the area year‐round (Käßmann & Woog, 2007). Since 2004, geese are ringed during molt catches (May and June), when adult geese are unable to fly. They are marked with a metal ring from the Vogelwarte Radolfzell on one leg and a blue plastic ring with white writing on the other leg. The main areas where they can be found within Stuttgart are the Inner City Parks (48°47′54″ N 9°12′24″ E) and the Max‐Eyth Lake (48°50′03″ N 9°12′55″ E), which are connected by the Neckar river. The geese move between the two areas, showing large variations in movement patterns (Käßmann & Woog, 2008).

Since 2018, with steadily increasing numbers of greylag geese across the state of Baden‐Württemberg, new breeding areas in nearby rural sites have been established by geese from different origins. One new breeding area is located at the protected area Zugwiesen (48°54′33″ N 9°15′24″ E), an artificially created wetland landscape with meadows and diverse wetland habitats. Large areas are protected by a fence and entry for humans is restricted. While the geese feed and roost within the protected area, they also use adjacent agricultural fields and meadows for feeding.

All geese received a unique goose ID to account for possible ring changes. Since 2007, weekly counts and ring readings have been conducted by the same observer. Consequently, many ringed greylag geese have a high number of resightings which allow a classification by their most‐frequented location (above 90% of resightings at this location). Greylag Geese observed predominantly within the two parks in the city area are considered urban, those observed at the Zugwiesen area are considered rural, based on the definition of Bourne and Simmons (1982). The city area including the Inner City Parks and Max‐Eyth Lake was defined as urban by the number of inhabitants per km^2^ (3040), while the inhabitants/km^2^ in the Zugwiesen area was lower with 611 individuals per km^2^ in the nearest settlement, some 200–300 m away (Poppenweiler).

Permits to catch and ring geese in Germany were obtained from the Regierungspräsidium Stuttgart and the Ministerium für Ernährung, Ländlichen Raum und Verbraucherschutz BW (permit numbers 31j‐9213.27/0004; 55‐6/8853.71; 55‐8853.17‐S; 55‐9213.47 and 55‐8841.03:8853.17). Blood samples were taken in accordance with national legislation with permits issued by the Regierungspräsidien Tübingen and Stuttgart (permit numbers 35‐9185.81/0391; 35‐9185.82/0223; RPS35‐9185.99/364; 55‐6/8853.71).

### 2.2 Laboratory analyses

Blood samples were collected from urban and rural greylag geese during the yearly catches. Samples were stored in buffer (EDTA‐Thymol‐NaF; Wink, 2006) at −20°C. Additional samples from autochthonous populations were used from the DNA sample collection of the State Museum of Natural History Stuttgart. For a list of all samples including accession numbers and genotype results, see Data Dryad (Sample Information file). A total of 322 greylag geese were sequenced and their DRD4 genotype was determined. Seventy‐five of the sampled greylag geese were from autochthonous populations, the remainder from the urban (*n* = 185) and rural areas (*n* = 76) in Germany (see Appendix S1, Table S1 for a list of locations and numbers of geese per genotype). DNA was extracted from 200 μL of the selected blood samples of urban and rural geese using the innuPREP Blood DNA Mini Kit (Analytik Jena). A 491 bp sequence of DRD4's exon 3 was amplified using published primers F1‐E3‐DR4D (5′‐CCRCTSAACTACAACCGGCG‐3′) and R1‐E3‐DR4D (5′‐YTCCCGGCCGTTGATCTTGG‐3′; Gillingham et al., 2012). PCR was carried out using the HotStarTaq Plus Master Mix Kit (Qiagen) in 20 μL reactions: 10 μL Master Mix, 2 μL 10× CoralLoad, 1 μL of each Primer (conc. 10 μM), 5 μL H_2_O and 1 μL of template DNA or a negative control (water). PCR was carried out under the following conditions: 5 min initial denaturation at 95°C; 35 cycles of 45 s denaturation at 95°C, 60 s annealing at 56°C and 60 s extension at 72°C; and a final extension step of 10 min at 72°C. PCR products were sequenced in both directions by LGC Genomics, Berlin and analyzed in Geneious 10.0.9 (https://www.geneious.com). Sequences were quality‐checked and analyzed visually by a single person for SNPs. Only SNPs detected in both forward and reverse sequences were counted for further analyses. In order to determine individual alleles, a total of 40 selected samples were cloned using the pGEM‐T‐Easy Vector Systems (Promega) according to the established protocol. Following Gillingham et al. (2012), seven clones were sequenced from each sample. Eleven alleles (*a*–*k*) were identified and submitted to GenBank (NCBI accession numbers ON502165 – ON502175). All geese with the genotypes *ad* and *bc* were among the 40 samples selected for cloning, as the two allele combinations produce identical bi‐allelic sequences. All other genotypes of non‐cloned geese could be inferred from combinations of the identified 11 alleles. Geneious 10.0.9 (https://www.geneious.com) was used to translate DNA sequences into protein sequences to determine if base substitutions were synonymous or non‐synonymous.

### 2.3 Field work

Flight Initiation Distances (FIDs) of geese were measured at the rural (Zugwiesen) and urban sites (Inner City Parks and Max‐Eyth Lake) simultaneously for a period of 1 year (May 2020 to May 2021). FIDs were collected using a standardized protocol: Ringed geese were slowly approached while grazing and three distances were measured (1) starting distance, (2) flight initiation distance (FID), and (3) the distance to the nearest water body (water distance). Distances were measured using a rangefinder (Nikon Prostaff 3i; Noblex Rangefinder NR 1000 and Bosch PLR 50 C). As four people performed the experiments, the observer was noted down for each measurement. To ensure that the geese would not get used to the procedure, there was a break of at least 1 day between measurements at the same location. While a total of 926 FID measurements were taken from 314 individuals, only measurements of geese with known genotypes were included in the statistical analyses. FIDs were measured for 156 geese whose genotypes were known. As most geese were measured more than once, the dataset contained 543 entries. To mitigate a possible seasonal effect on FID, all measurements throughout the seasons were included in the analysis. In total, 61 entries were from the rural locations (37 common and 24 rare), while the remaining 482 entries were from the urban locations (346 common and 136 rare).

### 2.4 Statistical analyses

Allele frequencies were calculated for geographically distinct goose populations (“Germany”, “Iceland”, “Norway”, “Denmark”, and “Greece”) using ARLEQUIN 3.5.2.1 (Excoffier & Lischer, 2010). “Germany” thereby refers only to the samples from the rural (Zugwiesen) and urban (Inner City Parks and Max‐Eyth Lake) areas. The urban and rural geese were tested for Hardy–Weinberg Equilibrium, allele frequencies were calculated and an analysis of molecular variance (AMOVA) was conducted to test for genetic differentiation between the two populations using ARLEQUIN 3.5.2.1 (Excoffier & Lischer, 2010). A Pearson's Chi‐squared test was performed in R 4.1.2 (R Core Team, 2019) run from R Studio (RStudio Team, 2021) to discover whether there is a difference in the allele frequencies between the German urban and rural populations (data in Data Dryad, Data File S1).

Due to the low numbers of the majority of the genotypes, they were categorized according to their frequency for further analyses, similar to van Dongen et al.'s (van Dongen et al., 2015) work. Two categories of genotype frequencies were defined based on all analyzed geese (*n* = 322) without taking location or FID measurements into account: common (occurring in more than 50 geese) and rare (occurring in 50 geese or less).

All following analyses were calculated in R 4.1.2 (R Core Team, 2019) run from R Studio (RStudio Team, 2021). Several Pearson's Chi‐squared tests were performed to discover whether there is a difference in genotype frequency between the locations, that is, whether geese with a specific genotype frequency occur more often in one location versus another. We used five subsets of all data to compare locations: (a) rural and urban geese; (b) rural, urban, and Icelandic geese; (c) rural, urban, and Norwegian geese; (d) rural, urban, and Danish geese and (e) rural, urban, and Grecian geese.

An ANOVA was performed to analyze the difference between FID in the urban and rural areas. The variation of the DRD4 genotype with individual FIDs in the German urban and rural areas was analyzed using the lme4 package, version 1.1.27.1 (Bates et al., 2015), and the lmerTest package, version 3.1‐3 (Kuznetsova et al., 2017). Flight initiation distances were not available for geese from autochthonous populations. A linear mixed model (LMM) was fitted using FID as response variable. Four explanatory variables were added as fixed effects based on van Dongen et al. (2015): location (urban or rural), genotype frequency (common or rare), starting distance, and water distance. Genotype frequency and location were added to the same model to avoid measuring a pseudo‐correlation. Starting distance was added as covariable to correct for it as suggested by Blumstein (2003) and water distance was added according to Guay et al. (2013). Flight initiation distance, starting distance, and water distance were log‐transformed (natural logarithm ln) prior to use to agree with assumptions of normality and homoscedasticity. As the FID of some geese was measured more than once, the Goose ID was added as random effect to control for pseudo‐replication. As four different observers measured FIDs, observer was also added as a random effect. REML was set to false in order to analyze fixed effects. A *t*‐test using Satterthwaite's method was used to assess whether the model coefficients significantly differed from zero. Significance was determined by *p*‐values, with *p* < .05 classified as significant.

## 3 RESULTS

Within the 491 bp sequence of DRD4, 10 SNPs were found. The majority of all SNPs were synonymous, with only three non‐synonymous SNPs. Eleven distinct alleles (*a*–*k*) were found, forming 35 genotypes. Two genotypes were most frequent (*aa*, *n* = 103 and *ab*, *n* = 89), while many genotypes were only found in a single individual (Table 1).

**TABLE 1:** List of genotypes and the overall number of geese carrying each genotype.

*Genotype*	Amount	*Genotype*	Amount	*Genotype*	Amount
*aa*	103	*bd*	4	*dh*	3
*ab*	89	*be*	1	*ee*	2
*ac*	8	*bf*	2	*ef*	1
*ad*	12	*bj*	1	*eh*	1
*ae*	8	*cc*	5	*ek*	1
*af*	3	*cd*	5	*fh*	1
*ag*	4	*ce*	4	*fi*	1
*ah*	3	cf	1	*fk*	1
*ai*	1	*ch*	3	*gg*	1
*aj*	5	*dd*	2	*hh*	1
*bb*	30	*de*	1	*jj*	1
*bc*	7	df	6		

**FIGURE 1:**
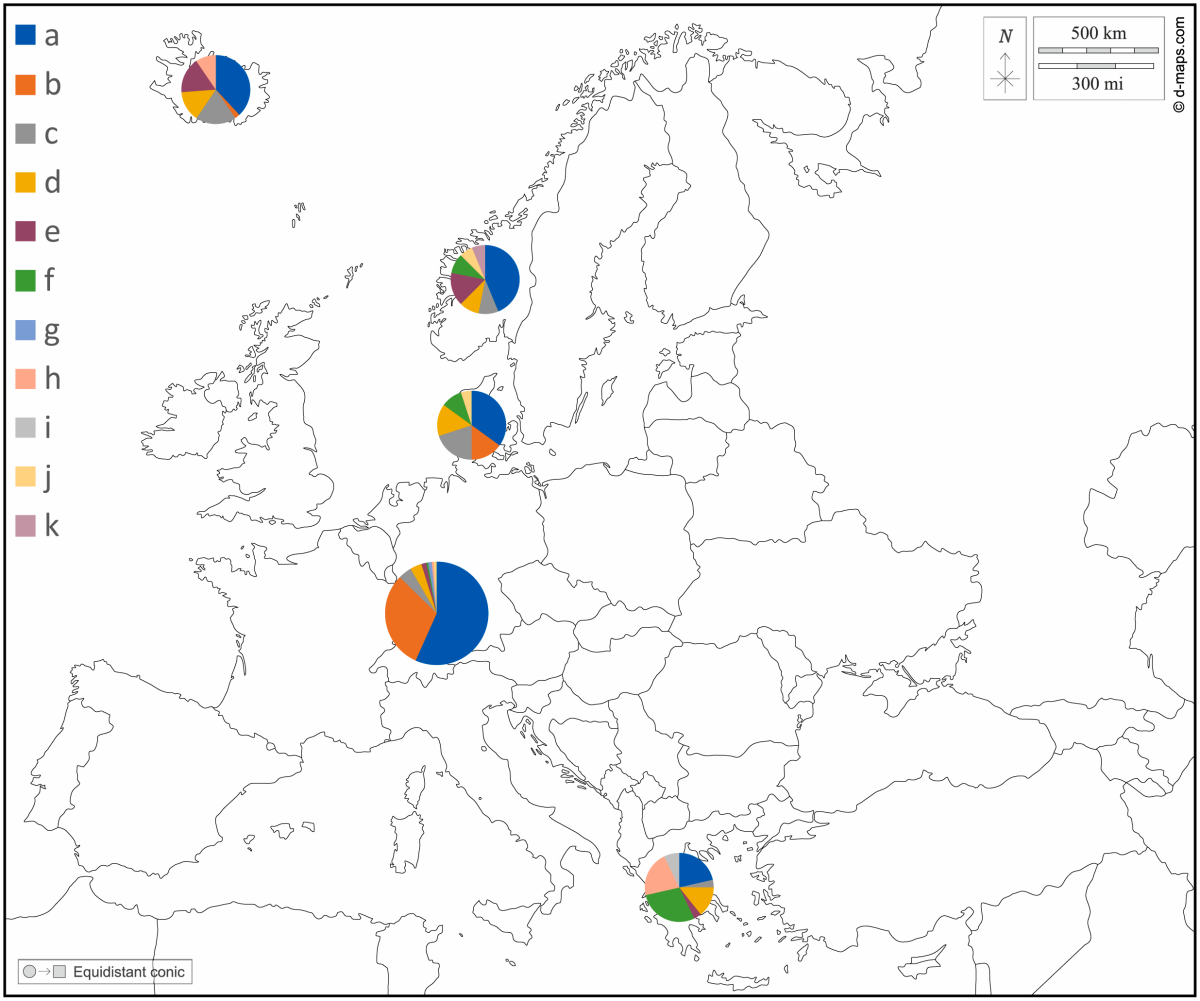
Frequency of DRD4 alleles in greylag geese in distinct European locations (from top left to bottom right: Iceland, Norway, Denmark, Germany, Greece). Background map from d‐maps.com, pie charts constructed in Microsoft Excel. Numbers underlying this graph can be found in the Appendix S1, Table S2.

Allele frequencies differed between geographically distinct populations (Figure 1), with some alleles being private either to the geese from Denmark, Norway, and Iceland (subspecies *A. a. anser*) or to geese from Greece (*A. a. rubrirostris*). In the geese from Germany, nine alleles were found, though alleles *i* and *k* were missing (Table 2 and Appendix S1, Table S2). Most alleles occurred at very low frequencies (<0.1; Table 2). Allele frequencies in geese from urban and rural areas were found to differ from each other (Table 3; *χ* ^2^ = 49.27, df = 8, *p* < .0001; *n* = 522). However, genetic differentiation between urban and rural geese was low (pairwise *F*_ST_ = .017, *p* = .021). Both urban and rural geese showed slight deviation from Hardy–Weinberg equilibrium. Observed heterozygosity (H_O_) of urban geese was 0.49, while expected heterozygosity (H_E_) was 0.54 (*p* = .00015). H_O_ of rural geese was 0.55, whereas H_E_ was 0.66 (*p* = .025).

**TABLE 2:** Allele frequencies of the 11 alleles *a*–*k* in the geographically distinct populations.

Allele	GER	DEN	ICL	NOR	GRC
*a*	0.57	0.35	0.38	0.44	0.21
*b*	0.31	0.15	0.02	0.00	0.00
*c*	0.04	0.20	0.19	0.09	0.04
*d*	0.04	0.15	0.14	0.09	0.14
*e*	0.02	0.00	0.17	0.16	0.04
*f*	0.01	0.10	0.00	0.09	0.29
*g*	0.01	0.00	0.00	0.00	0.00
*h*	0.01	0.00	0.10	0.00	0.21
*i*	0.00	0.00	0.00	0.00	0.07
*j*	0.01	0.05	0.00	0.06	0.00
*k*	0.00	0.00	0.00	0.06	0.00

Abbreviations: DEN, Denmark; GER, Germany (from urban and rural areas); GRC, Greece; ICL, Iceland; NOR, Norway.

**TABLE 3:** Allele frequencies of the 11 alleles *a*–*k* within GER animals (urban vs. rural).

Allele	Urban	Rural
*a*	0.59	0.51
*b*	0.33	0.25
*c*	0.02	0.09
*d*	0.01	0.09
*e*	0.02	0.01
*f*	0.01	0.00
*g*	0.00	0.03
*h*	0.00	0.01
*i*	0.00	0.00
*j*	0.01	0.00
*k*	0.00	0.00

Across all analyzed samples, common genotypes (*aa* and *ab*) were found in 192 individuals, while 130 geese had rare genotypes (all 33 other genotypes). In the German geese, which occur in an urban and a rural area, common genotypes occurred more often in urban areas and rare genotypes more often in rural areas (*χ* ^2^ = 7.98, df = 1, *p* = .005; *n* = 261). When comparing samples from the urban and rural areas (in Germany) with samples from Iceland, common genotypes occurred less often in Iceland, and rare genotypes were more frequent (*χ* ^2^ = 25.57, df = 2, *p* < .0001; *n* = 282). Comparing samples from the urban and rural areas with the sample set from Norway (*χ* ^2^ = 21.28, df = 2, *p* < .0001; *n* = 277), Denmark (*χ* ^2^ = 15.12, df = 2, *p* = .0005; *n* = 271), or Greece (*χ* ^2^ = 30.59, df = 2, *p* < .0001; *n* = 275) resulted in the same pattern.

**FIGURE 2:**
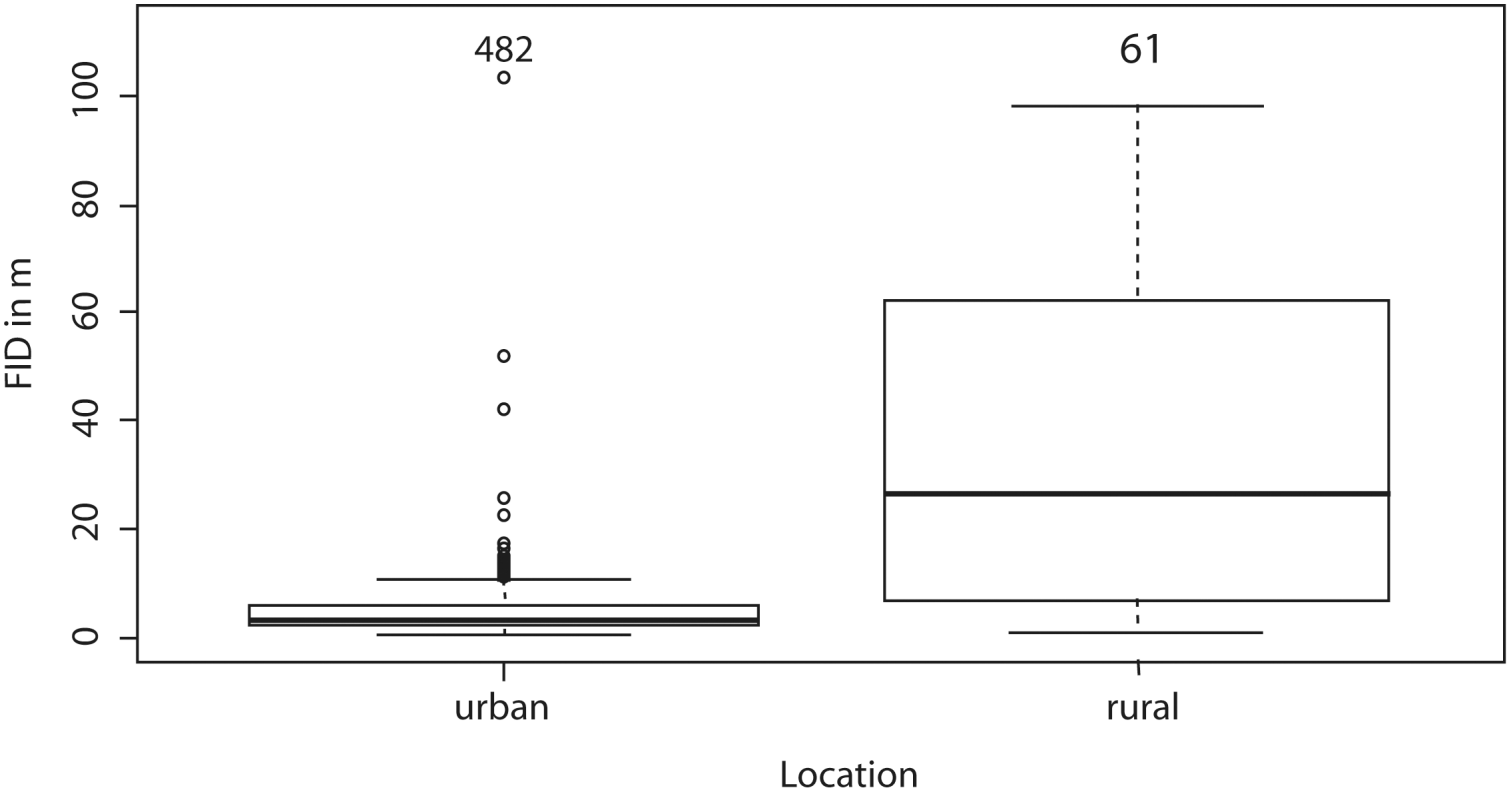
FIDs of geese measured in the urban and rural areas.

FID was significantly lower in the urban area, averaging less than 10 m, and higher in the rural area (Figure 2, ANOVA *p* < .0001). The same pattern could be confirmed in the linear mixed model (Table 4; effect of Location on FID *p* < .0001; estimate −1.403, SE 0.12, df 409.653, *t*‐value −11.691). The linear mixed model showed that FIDs were higher when the starting distance was higher (*p* < .0001, estimate 0.499, SE 0.055, df 521.155, *t*‐value 9.125). No effect of water distance (*p* = .266, estimate 0.028, SE 0.025, df 537.657, *t*‐value 1.113) or genotype frequency (*p* = .911, estimate −0.009, SE 0.08, df 122.947, *t*‐value −11.691) on FID could be detected (Table 4).

**TABLE 4:** Linear model on the response variable FID (flight initiation distance) showing the effect of location (urban vs. rural), genotype frequency (common vs. rare), starting distance (log), and distance to the nearest water body (log).

Flight initiation distance					
Fixed effects					
Variables	Estimate	Standard error	Degrees of freedom	*t* value	*p* value
(Intercept)	0.715	0.285	65.367	2.541	.006**
Location—urban	−1.403	0.120	409.653	−11.691	<.0001***
Genotype frequency—rare	−0.009	0.080	122.947	−0.113	**.911 n.s.**
Log of starting distance	0.499	0.055	521.155	9.125	<.0001***
Log of distance to nearest water body	0.028	0.025	537.657	1.113	**.266 n.s.**

Random effects		
Group	Variance *σ* ^2^_*ε*_	Standard deviance *σ*
GooseID (Intercept)	0.049	0.221
Observer (Intercept)	0.067	0.259
Residual	0.495	0.703

*Note*: Goose ID and observer were added as random effects. FID measurements (*n* = 543); goose IDs (*n* = 156); observers (*n* = 4). Significance of *p* values: n.s. (non significant), ** (<.01), *** (<.001).

## 4 DISCUSSION

In this study, we describe the variability of the DRD4 gene in greylag geese in Europe and compare an urban and a rural population in Germany. To explore a possible link between this genetic background and risk‐prone behavior (boldness), we additionally analyzed whether genotype frequencies of DRD4 are associated with FID as a measure of boldness, which has only been done in a few other avian species (Holtmann et al., 2016; van Dongen et al., 2015). While there was a difference in genotype and allele frequencies in the urban and rural populations, no association with FID was detected.

In Greylag geese, the analyzed fragment of the DRD4 gene had 10 variable sites (=SNPs) resulting in 11 DRD4 alleles and 35 genotypes. Due to the large number of different genotypes, many only occurred in a single goose. Therefore, we were faced with similar power issues as van Dongen et al. (2015) despite analyzing a higher sample size. In their study on black swans, only six SNPs, five alleles, and five genotypes were found (van Dongen et al., 2015). However, van Dongen et al. (2015) studied two small local populations, likely documenting only a smaller part of the DRD4 diversity. To minimize a possible downward bias in estimating SNP numbers (Morin et al., 2004), we additionally sequenced greylag geese from several autochthonous populations. Most of the SNPs found were synonymous, but no distinction was made between types of base‐pair substitutions in subsequent analyses. First, it is possible that the protein function is changed by synonymous SNPs, for example through aberrant mRNA splicing or stability issues, which may be targeted by natural selection (Sauna & Kimchi‐Sarfaty, 2011). Second, synonymous SNPs may be linked to other regions within DRD4 which may have an effect on protein functionality (Gillingham et al., 2012; see Hunt et al., 2009 for an overview). Similarly, SNPs may be linked to other genes. This is the case in domestic chicken (*Gallus gallus domesticus*), where DRD4 haplotypes have been associated with feather pecking through linkage disequilibrium, which extends into a neighboring gene (Flisikowski et al., 2009).

Two genotypes, *aa* and *ab*, were most frequent across all populations, with allele *a* also being the most frequent allele. The high frequency of the allele *a* suggests some selective benefit of this allele (Hill et al., 1991; Morjan & Rieseberg, 2004; Rieseberg & Burke, 2001; Trachtenberg et al., 2003). The unequal frequencies of alleles and genotypes align with data from great tits (Fidler et al., 2007; Korsten et al., 2010), greater flamingos (*Phoenicopterus roseus*; Gillingham et al., 2012), common waxbills (*Estrilda astrild*; Carvalho et al., 2013), and collared flycatchers (Garamszegi et al., 2014). Greylag geese from Germany hold nine different alleles, including one private allele not detected in the autochthonous populations (*g*), and thus showed the highest diversity among our study populations. This may be due to the high numbers of geese analyzed, but it may also be due to the genetic variability of the founding animals that consisted of released individuals and escapees from both subspecies, *A. a. anser* and *A. a. rubrirostris*. Compared to the autochthonous populations, allele frequencies were very low for alleles *c* – *k* (hereafter rare alleles). The founding effect at the establishing of our local populations and subsequent genetic drift may have led to a loss of rare alleles from the populations (Nei et al., 1975). Alternatively, there may have been a selection process, with only animals with common alleles being able to successfully live and reproduce in urban areas (Møller, 2009). Allele frequencies in the autochthonous populations appeared to be more evenly distributed. Unfortunately, for these populations, we were only able to sequence a very limited number of animals (between *n* = 10 and *n* = 21). Sequencing more individuals may lead to changed allele frequencies for these populations. However, the presence of rarer alleles in our small sample size of autochthonous populations indicates they may not be rare overall. Despite differing allele frequencies, genetic differentiation at the DRD4 locus between the urban and rural populations was low. Both urban and rural geese populations deviated slightly from Hardy–Weinberg equilibrium, likely effected by our small sample size and the likely movement of individuals in or out of the populations (Alghamdi & Padmanabhan, 2014).

Common genotypes were significantly more frequent in urban areas, while rare genotypes occurred more often in rural areas. This distribution may be due to behavioral differences between the urban and rural geese (Møller, 2009). Among other functions, the dopaminergic system controls fear (Falzone et al., 2002; Garpenstrand et al., 2001). As a higher fear threshold is necessary for birds living in urban areas (Carrete & Tella, 2011; Møller, 2009), common genotypes may be correlated to higher fear thresholds and a bolder personality. We therefore measured FID as a function of wariness in birds (Blumstein, 2006). In urban areas, FID was significantly lower than in the rural areas. This is comparable to data from other species such as song sparrows *Melospiza melodia*, where urban animals were bolder and had a lower FID than rural animals (Scales et al., 2011). No significant effect of genotype frequency on FID could be detected. The model detected an effect of location on FID, as already shown in the chi‐square tests. However, location and genotype frequency could not be separated into different models, as including only one variable would risk measuring a pseudo‐correlation (i.e., seeing a genotype effect but in reality measuring a location effect). The model includes very few rural FID measurements (61, in comparison to 482 urban measurements). In part, this was due to the difficulty of measuring FIDs at the rural sites, where often only one measurement a day was possible. This was caused by occasional difficulties in finding the geese, but also because FIDs were only measured from relaxed geese. Once a single measurement was taken at a rural site, geese often flew away to the nearest water body. Geese with rare genotypes but no FID measurements as a result of this difficulty could not be included in the model. Additionally, due to a lack of DNA samples, the DRD4 genotype could not be determined for all geese with FID measurements. The rural area is a wide‐spread reserve where entry for humans is restricted, thus not all geese could be caught and sampled during the yearly catches. These two points combined may explain why there is no clear correlation of location with genotype frequency in the model, where only geese with both a known genotype and known FIDs were included. It may also be possible that the effect of genotype on FID is diluted by the grouping of genotypes into common and rare. However, many of the genotypes occurred in only one goose, which is statistically difficult to analyze. Human presence is very common in all Stuttgart parks, and occasionally some pedestrians will stop to feed the geese (S Mai, pers. obs.). Habituation of the geese (as defined in Rankin et al., 2009) to the constant human presence can therefore not be discounted. The different allele and genotype frequencies between urban and rural habitats in our data do not support this, but Cooke (1980) suggested that birds in urban habitats have more opportunities to learn when humans become a danger (Carrete & Tella, 2011; Cooke, 1980). While no individual habituation to human presence has been detected in burrowing owls (*Athene cunicularia*; Carrete & Tella, 2010), it is likely that this varies between species (Carrete & Tella, 2011). Indeed, in some species, their behavior changes in urban habitats and can be linked to behavioral flexibility (Sol et al., 2013). In western fence lizards (*Sceloporus occidentalis*), for example, different FIDs between an urban and a rural site were attributed to the different exposure to people (Grolle et al., 2014).

Apart from its effect on wariness, DRD4 has been associated with exploratory behavior (Fidler et al., 2007; Mueller et al., 2013), novelty seeking (Bailey et al., 2007; Dulawa et al., 1999; Garamszegi et al., 2014; Mueller et al., 2014; Riyahi et al., 2017; Timm et al., 2015), escape behavior (Kluen et al., 2012), or body condition (Gillingham et al., 2012). In burrowing owls, rural owls were more fearful of humans, as well as less explorative and less aggressive (Carrete & Tella, 2017). Greylag geese with common genotypes may possibly show more exploratory behavior. While the initial colonization of Stuttgart is based on a few escaped geese, more geese arrived from beyond the city limits over the years (Hölzinger et al., 2004; Woog et al., 2008). It is possible that those were bolder animals exploring new habitats. As geese with common genotypes occur more often in urban areas, bolder animals could be those carrying common genotypes. In yellow‐crowned bishops, DRD4 variation is related to differences in neophobic behavior in the early stages of invasion (Mueller et al., 2017). This would hint toward pre‐establishment selection, where animals with specific traits or personalities are more likely to, for example, escape captivity (Carrete et al., 2012; Chapple et al., 2012). Compared to a shy individual, a bold animal would be more likely to escape when give a chance (Carrete et al., 2012). Explorative behavior can also be an advantage, both to escape captivity and in early stages of invasion. For example, in newly established populations of invading house sparrows, neophobia is lower than in old resident populations, which may explain the species' success in urban areas (Martin & Fitzgerald, 2005).

### 4.1 SUMMARY AND OUTLOOK

In our study, we showed that greylag geese had a high number of variable sites in DRD4's exon 3. The 11 alleles and 35 genotypes showed a skewed distribution, with two genotypes occurring more frequently than the remaining 33. When comparing urban and rural populations of greylag geese living in and near Stuttgart, we found clearly differing allele and genotype frequencies. Greylag geese with common genotypes occurred more often in urban areas, hinting toward a selective benefit in urban animals. While the differing genotype frequencies could not be correlated with FID as a measure of the geese's shyness or boldness, this may be due to the small sample sizes in rural areas. Overall, our data associates common DRD4 genotypes in greylag geese with urban areas. It presents a good starting point for further studies analyzing the rural area in‐depth or adding other candidate genes to the analysis.

## AUTHOR CONTRIBUTIONS

**Sabrina Mai:** Conceptualization (equal); data curation (equal); formal analysis (lead); funding acquisition (equal); investigation (equal); methodology (lead); project administration (equal); software (equal); visualization (lead); writing – original draft (lead); writing – review and editing (lead). **Caroline Wittor:** Conceptualization (supporting); data curation (equal); formal analysis (supporting); investigation (equal); methodology (supporting); software (equal); visualization (supporting); writing – original draft (supporting); writing – review and editing (supporting). **Stefan Merker:** Conceptualization (supporting); formal analysis (supporting); funding acquisition (equal); methodology (supporting); resources (equal); supervision (supporting); visualization (supporting); writing – original draft (supporting); writing – review and editing (supporting). **Friederike Woog:** Conceptualization (equal); formal analysis (supporting); funding acquisition (equal); methodology (supporting); project administration (equal); resources (equal); supervision (lead); visualization (supporting); writing – original draft (supporting); writing – review and editing (supporting).

## BENEFIT‐SHARING STATEMENT

The Nagoya Protocol is not applicable because samples were obtained prior to the 12th October 2014 thus no benefit sharing obligations apply. Greylag geese are a huntable species in all countries samples were obtained from.

## Supporting information


Appendix S1

## Data Availability

The genetic data generated by this study (DRD4 alleles) are accessible at GenBank with the accession numbers ON502165‐ON502175. The data underlying the study and the R‐script can be found at Data Dryad (https://doi.org/10.5061/dryad.z34tmpgj1). The following files are uploaded there: an excel file with a list of all analyzed greylag geese, including museum accession numbers (SampleInformation.xlsx); six csv data files (S1 to S6) as input for R for chi‐square tests (DataFile_S1_Alleles.csv and following); one csv data file as input for R for the model (DataFile_S7_Model.csv); one R script containing all analyses (Script_RStudio.txt); and one Readme file with additional explanations.
