# Supplementary material for: DRD4 allele frequencies in greylag geese vary between urban and rural sites
Source: Ecol Evol. 2023 Feb 8;13(2):e9811. doi: 10.1002/ece3.9811 (PMC9909002; doi:10.1002/ece3.9811)
Supplement: Supplementary file 1 — Appendix S1 [file ECE3-13-e9811-s001.docx]

**Appendix**

Table S1. Number of geese with each genotype according to location. GER, Germany (urban and rural); DEN, Denmark; ICL, Iceland; NOR, Norway; GRC, Greece.

| **Genotype** | **GER** | **DEN** | **ICL** | **NOR** | **GRC** |
| --- | --- | --- | --- | --- | --- |
| *aa* | 92 | 2 | 4 | 4 | 1 |
| *ab* | 87 | 1 | 1 | 0 | 0 |
| *ac* | 4 | 1 | 2 | 1 | 0 |
| *ad* | 11 | 0 | 1 | 0 | 0 |
| *ae* | 2 | 0 | 3 | 3 | 0 |
| *af* | 1 | 0 | 0 | 0 | 2 |
| *ag* | 4 | 0 | 0 | 0 | 0 |
| *ah* | 1 | 0 | 1 | 0 | 1 |
| *ai* | 0 | 0 | 0 | 0 | 1 |
| *aj* | 2 | 1 | 0 | 2 | 0 |
| *bb* | 29 | 1 | 0 | 0 | 0 |
| *bc* | 7 | 0 | 0 | 0 | 0 |
| *bd* | 4 | 0 | 0 | 0 | 0 |
| *be* | 1 | 0 | 0 | 0 | 0 |
| *bf* | 2 | 0 | 0 | 0 | 0 |
| *bj* | 1 | 0 | 0 | 0 | 0 |
| *cc* | 3 | 1 | 1 | 0 | 0 |
| *cd* | 2 | 0 | 2 | 1 | 0 |
| *ce* | 1 | 0 | 2 | 1 | 0 |
| *cf* | 0 | 1 | 0 | 0 | 0 |
| *ch* | 2 | 0 | 0 | 0 | 1 |
| *dd* | 1 | 1 | 0 | 0 | 0 |
| *de* | 0 | 0 | 1 | 0 | 0 |
| *df* | 0 | 1 | 0 | 2 | 3 |
| *dh* | 0 | 0 | 2 | 0 | 1 |
| *ee* | 2 | 0 | 0 | 0 | 0 |
| *ef* | 0 | 0 | 0 | 0 | 1 |
| *eh* | 0 | 0 | 1 | 0 | 0 |
| *ek* | 0 | 0 | 0 | 1 | 0 |
| *fh* | 0 | 0 | 0 | 0 | 1 |
| *fi* | 0 | 0 | 0 | 0 | 1 |
| *fk* | 0 | 0 | 0 | 1 | 0 |
| *gg* | 1 | 0 | 0 | 0 | 0 |
| *hh* | 0 | 0 | 0 | 0 | 1 |
| *jj* | 1 | 0 | 0 | 0 | 0 |
|  |  |  |  |  |  |
| **Number of geese** | **261** | **10** | **21** | **16** | **14** |

Table S2. Number of alleles within each location. Absence of the allele (0 geese) is highlighted in red. GER, Germany (urban and rural); DEN, Denmark; ICL, Iceland; NOR, Norway; GRC, Greece.

| **Allele** | **GER** | **DEN** | **ICL** | **NOR** | **GRC** |
| --- | --- | --- | --- | --- | --- |
| *a* | 296 | 7 | 16 | 14 | 6 |
| *b* | 160 | 3 | 1 | 0 | 0 |
| *c* | 22 | 4 | 8 | 3 | 1 |
| *d* | 19 | 3 | 6 | 3 | 4 |
| *e* | 8 | 0 | 7 | 5 | 1 |
| *f* | 3 | 2 | 0 | 3 | 8 |
| *g* | 6 | 0 | 0 | 0 | 0 |
| *h* | 3 | 0 | 4 | 0 | 6 |
| *i* | 0 | 0 | 0 | 0 | 2 |
| *j* | 5 | 1 | 0 | 2 | 0 |
| *k* | 0 | 0 | 0 | 2 | 0 |
